# Adherence, Disease Control, and Misconceptions Related to the Use of Inhalation Therapy in Patients with Obstructive Pulmonary Diseases: A Cross-Sectional Study

**DOI:** 10.3390/medicina60060853

**Published:** 2024-05-23

**Authors:** Dejan Živanović, Jovan Javorac, Dejana Savić, Andrijana Mikić, Marija Jevtić, Miroslav Ilić, Violeta Kolarov, Ivana Minaković, Bela Kolarš, Mirjana Smuđa, Vesna Mijatović Jovin

**Affiliations:** 1 Faculty of Medicine, University of Novi Sad, 21000 Novi Sad, Serbia; dejan.zivanovic@mf.uns.ac.rs (D.Ž.); 014857@mf.uns.ac.rs (D.S.); mirjana.smudja@uns.ac.rs (M.S.); 2 Department of Psychology, College of Human Development, 11000 Belgrade, Serbia; andrijana.mikic@ahr.edu.rs; 3 Department of Internal Medicine, Faculty of Medicine, University of Novi Sad, 21000 Novi Sad, Serbia; miroslav.ilic@mf.uns.ac.rs (M.I.); violeta.kolarov@mf.uns.ac.rs (V.K.); 4 Institute for Pulmonary Diseases of Vojvodina, 21204 Sremska Kamenica, Serbia; 5 Department of Hygiene, Faculty of Medicine, University of Novi Sad, 21000 Novi Sad, Serbia; marija.jevtic@uns.ac.rs; 6 Institute of Public Health of Vojvodina, 21000 Novi Sad, Serbia; 7 Research Center on Environmental and Occupational Health, School of Public Health, Université Libre de Bruxelles (ULB), 1050 Brussels, Belgium; 8 Department of General Medicine and Geriatrics, Faculty of Medicine, University of Novi Sad, 21000 Novi Sad, Serbia; ivana.minakovic@mf.uns.ac.rs (I.M.); bela.kolars@mf.uns.ac.rs (B.K.); 9 Health Center “Novi Sad”, 21000 Novi Sad, Serbia; 10 Department of Higher Medical School, Academy of Applied Studies Belgrade, 11000 Belgrade, Serbia; 11 Department of Pharmacology, Toxicology and Clinical Pharmacology, Faculty of Medicine, University of Novi Sad, 21000 Novi Sad, Serbia; vesna.mijatovic-jovin@mf.uns.ac.rs

**Keywords:** asthma, COPD, adherence, inhalation therapy, misconceptions, disease control

## Abstract

*Background and Objectives*: Inadequate treatment of asthma and chronic obstructive pulmonary disease (COPD) might have a negative impact on their progression. Inhalation therapy is the cornerstone of pharmacotherapy for these conditions. However, challenges such as low adherence, negative attitudes, and misconceptions about inhaled medications still persist, impeding effective disease management. This study aimed to evaluate adherence, ascertain the level of disease control in asthma and COPD, explore potential misconceptions surrounding inhalation therapy among patients with obstructive lung diseases and the general population in Vojvodina, and evaluate the reliability of newly developed questionnaires employed in the study. *Materials and Methods*: This cross-sectional study utilized a battery of questionnaires encompassing sociodemographic data, the Asthma Control Test (ACT), the COPD Assessment Test (CAT), along with two novel questionnaires—one for assessing adherence and another for analyzing attitudes toward inhalation therapy. Statistical analyses were conducted using SPSS software, version 25.0. *Results*: The average ACT score among patients with asthma was 17.31, while it was 19.09 for the CAT questionnaire among COPD patients. The composite score on the newly developed adherence assessment questionnaire was 2.27, exhibiting a reliability coefficient lower than recommended (α = 0.468). Significant statistical differences emerged among sample subgroups regarding attitudes and misconceptions toward inhalation therapy. The reliability coefficient for this questionnaire was deemed satisfactory (α = 0.767). *Conclusions*: Adherence rates were notably suboptimal in both subgroups of the studied population. The disease control levels were higher among asthma patients, while they exhibited less prevalent misconceptions regarding inhalation therapy compared to COPD patients and the healthy population.

## 1. Introduction

According to the current GOLD recommendations, chronic obstructive pulmonary disease (COPD) is defined as a heterogeneous lung condition characterized by chronic respiratory symptoms (dyspnea, cough, sputum production, and/or exacerbations) due to abnormalities in the airways (bronchitis and bronchiolitis) and/or alveoli (emphysema) that cause persistent, often progressive, irreversible airflow obstruction [1]. Considering the high prevalence and mortality rates, as well as the significant economic costs associated with treating these patients, today, COPD is one of the leading global public health issues [2,3]. COPD is associated with abnormal inflammatory responses of the lungs to harmful particles and gases. Although cigarette smoking is the primary cause of COPD, up to 25% of affected individuals have never smoked. Prolonged exposure to other lung irritants, such as air pollution, chemical fumes, and dust (especially in the workplace), can also contribute to the development of COPD. Additionally, a rare genetic condition called α1-antitrypsin deficiency may also be a cause of this disease [4].

Asthma is defined by the current GINA recommendations as a heterogeneous disease with chronic inflammation of the airways, respiratory symptoms (such as coughing, wheezing, shortness of breath, and chest tightness) that change in intensity over time, and variable airflow limitation during expiration [5]. Variable airflow obstruction is usually reversible, either spontaneously or with pharmacological treatment, albeit in certain people the reversibility may not be complete [6]. Viral infections, tobacco smoke, physical activity, stress, and allergens from the home and workplace (such as house dust mites, pollen, and cockroach droppings) can all cause or exacerbate asthma symptoms. Asthma exacerbations can also be caused by the use of certain medications, such as β-blockers, aspirin, or other nonsteroidal anti-inflammatory drugs (NSAIDs) [5].

Using inhalation therapy is the cornerstone of treating COPD and asthma: β2 agonists are used for bronchodilation, while inhaled corticosteroids (ICS) are used for long-term reduction of airway inflammation [7,8]. According to the GOLD recommendations, the foundation of pharmacological treatment for stable COPD involves the use of long-acting β2-agonists and/or anticholinergics (LABA or LAMA) as maintenance therapy. The addition of inhaled corticosteroids (ICS) to therapy can be considered in patients with frequent COPD exacerbations and high levels of blood eosinophils [1]. On the other hand, according to GINA recommendations, the preferred approach to pharmacological asthma treatment involves the use of ICS in combination with formoterol for maintenance and reliever therapy (single maintenance and reliever treatment, SMART) [5]. Currently, there are various devices available for inhalation therapy, including pressurized meter-dosed inhalers (pMDIs), dry powder inhalers (DPIs), breath-actuated inhalers, soft-mist inhalers (SMIs), and nebulizers. All these devices are clinically equivalent, and the appropriate choice of inhaler is an important factor in optimizing inhalation therapy outcomes [7]. Developing proficiency in inhaler use is essential for managing COPD and asthma. While there are many benefits to inhalation therapy, patients must also be well versed in how to use these devices correctly. Unfortunately, despite the availability of modern devices, improper inhaler use remains common in the daily lives of patients with obstructive lung diseases, regardless of the type of inhaler used and the type of disease. Proper education by healthcare professionals will undoubtedly help patients master the inhalation technique, and in this regard, it is crucial to build a positive therapeutic relationship between the prescribing physician and the nurse educating the patient, as well as between the patient and their family. By improving or developing innovative technologies that could considerably limit the incorrect use of inhalation drugs, manufacturers of inhalation therapy equipment can also help further in reaching this goal [9,10,11].

Medication adherence (synonym: compliance) refers to the extent to which a person takes medications as prescribed [12]. Adherence is one of the main challenges encountered in the treatment of many chronic diseases, as it represents the link between the effectiveness of the treatment regimen and disease management. High adherence is a crucial factor for the effective implementation of inhalation therapy in the treatment of asthma and COPD patients. Low levels of control of these diseases, as well as higher rates of absenteeism from work and school, irrational use of healthcare services, and higher rates of morbidity and mortality, are all strongly correlated with inadequate patient cooperation regarding the regular use of inhalation therapy [12,13]. However, the results of previous research worldwide often indicate a low level of patient awareness regarding the method of administration, therapeutic effects, and potential side effects of inhalation medications [14,15]. As a result of insufficient awareness, negative attitudes and misconceptions toward regular inhalation therapy are not uncommon in the population of patients with obstructive pulmonary diseases: patients often cite fear of the addictive potential of inhalation medication and fear of possible adverse effects of the medication [15,16,17,18,19], but negative perception can sometimes lead to a loss of independence or discomfort in social situations [20]. According to literature sources, the most prevalent concern associated with the side effects of inhalation drugs is that of corticosteroid preparations. In some cases, this anxiety can even be so intense as to resemble a phobia [21,22]. Adherence-related issues are one of the most significant barriers to the effective implementation of inhalation therapy. As can be seen from the literature sources used, this problem has been present in the treatment of pulmonary patients for many years [15,17,22], but recent research confirms that it is still globally relevant [16,20].

Considering the above, this study aimed to assess adherence, disease control level, attitudes, and potential misconceptions regarding the use of inhalation therapy among patients with asthma and COPD, as well as the correlation between the mentioned variables. In order to determine the real prevalence of misconceptions regarding the use of this type of medication, we also examined the attitudes regarding the use of inhalation therapy among individuals from the general population who are not afflicted with respiratory disorders. Furthermore, a statistical test was performed to determine the reliability of two newly created questionnaires that were used in this study: the Adherence with Inhaler Therapy Questionnaire (AITQ) and the Inhaler Therapy Attitudes Questionnaire (ITAQ).

## 2. Materials and Methods

The research was conducted between November and December 2023 as a cross-sectional observational study. Two distinct cohorts were examined: The first cohort (Group A) comprised patients diagnosed with asthma and COPD receiving treatment at the Institute for Pulmonary Diseases of Vojvodina in Sremska Kamenica, Serbia. The second cohort (Group B) consisted of individuals from the general population residing in the Serbian Autonomous Province of Vojvodina who were not under treatment for respiratory conditions. The determination of the sample size for this study was based on several factors, including feasibility, the desired level of statistical power, resource availability, and the specific aims of the research. The inclusion of respondents in the study was on a voluntary basis, and all respondents signed informed consent on a previously prepared form before entering the research. 

As a research instrument, a battery of questionnaires was utilized, consisting of the questionnaire on the sociodemographic characteristics of participants relevant to the study, as well as available and previously Serbian language-validated questionnaires for assessing asthma symptom control—the Asthma Control Test (ACT) and COPD symptom control—COPD Assessment Test (CAT). Additionally, a newly developed questionnaire was utilized to evaluate inhalation therapy adherence, and a second newly developed questionnaire was utilized to assess attitudes regarding this type of therapy. While participants in Group B only completed the questionnaire on demographic data and the questionnaire on attitudes toward the use of inhalation therapy, participants in Group A were required to complete all of the aforementioned questionnaires. The sociodemographic data questionnaire comprised questions related to age, gender, occupation, level of formal education, employment status, monthly income, marital status, place of residence, and existing comorbidities. Within this questionnaire, patients from Group A also provided responses regarding the type of diagnosed obstructive respiratory disease (asthma or COPD), the date of disease diagnosis, the number of hospitalizations due to exacerbations of the diagnosed obstructive respiratory disease, information on the regularity of prescribed inhalation medication intake, as well as details concerning the therapy currently used by the participant. The ACT is employed to evaluate the level of asthma control and consists of five questions. Respondents can score between a minimum of 5 and a maximum of 25 points on this questionnaire. A score falling within the range of 20–25 points is indicative of well-controlled asthma. Conversely, scores between 16 and 19 points suggest poorly controlled asthma, while scores in the range of 5 to 15 points indicate very poorly controlled asthma. The CAT is designed to evaluate how COPD affects a patient’s daily life and overall health. It consists of a total of eight questions. The minimum possible score on this questionnaire is 0, while the maximum score is 40. It is considered that COPD has a minimal (small) impact on a patient’s life if the total score achieved by the respondent is less than 10. A moderate impact is indicated if the score ranges from 10 to 20, a large impact if the score achieved is between 21 and 30, and a very strong impact if the score exceeds 30. The newly constructed AITQ is intended to evaluate the extent to which respondents adhere to the instructions for inhalation therapy provided by therapists. Respondents from Group A completed the 10 questions by selecting 1 of the 4 provided responses (usually/often, sometimes, rarely, or never/not at all), achieving an item score ranging from 0 to 3. This questionnaire has a minimum score of 0, which denotes extremely low adherence, and a maximum score of 30, which denotes good adherence. For some questions in the AITQ, a 3-month time period was selected to capture adherence behaviors and experiences over a sufficiently meaningful period while minimizing recall bias. This duration was previously used in some research assessing medication adherence and has been shown to be effective in capturing trends and patterns in adherence behaviors over time [23]. The newly constructed ITAQ was developed for this study as an instrument to assess respondents’ attitudes towards the use of inhalation therapy. It comprises 20 statements, and respondents from both groups were asked to indicate their agreement with each individual statement by choosing one of the provided responses on a five-point Likert scale (1—strongly disagree, 2—mostly disagree, 3—neither agree nor disagree, 4—mostly agree, or 5—strongly agree), resulting in a score ranging from 1 to 5 for each item. As previously mentioned, the ACT and CAT questionnaires have been previously validated in earlier studies and have demonstrated a satisfactory level of reliability [24,25]. The reliability of the newly developed questionnaires was tested statistically within the scope of this study.

The part that involved interviewing patients with asthma and COPD was carried out at the Institute for Pulmonary Diseases of Vojvodina either during patients’ hospital stays or as part of outpatient exams at the Polyclinic Department of the Institute. The ITAQ and the modified sociodemographic data questionnaire were hosted on the Google Forms platform with the aim of surveying the healthy population. The preamble of the questionnaire was customized for this purpose and contained all relevant study information. The questionnaire link was distributed via social networks, and respondents provided informed consent for voluntary participation in the study by clicking the “I Accept” button.

The statistical analysis of the obtained research results was conducted using SPSS (Statistical Package for the Social Sciences for Windows, version 25.0) and included the application of various methods of descriptive statistics and hypothesis testing. First, the reliability of the questionnaire was examined as a metric property of the tests by determining the value of the Cronbach’s alpha coefficient (α), which measures internal consistency. Depending on the nature of the analyzed parameters, frequencies, percentages, and the mean or arithmetic mean (M) were used to describe significant parameters. The standard deviation (SD) was used as a measure of deviation from the mean. The minimum and maximum sample values of numerical variables were also presented in the results. The type of statistical test applied was determined by the type of collected data (categorical or numerical variables). A one-way analysis of variance (ANOVA) was used to test differences between parameters. In cases where statistically significant differences between modalities of a categorical variable were identified, the Tukey post-hoc test was used to compare the average values of a numerical variable across all modalities of the qualitative variable. Differences between two modalities of a qualitative variable and a numeric variable were tested using the independent sample *t*-test. The chi-square test was used to examine the relationship between two categorical variables. The statistical significance of the obtained results was interpreted at the level of *p* < 0.05.

## 3. Results

### 3.1. Sociodemographic Data

The study included a total of 180 participants, of whom 47 (26.1%) were patients diagnosed with asthma, 33 (18.3%) were patients diagnosed with COPD, while 100 participants (55.6%) constituted the sample without a diagnosis of respiratory disease (the sample of healthy participants). The average age of the entire sample was 51.5 years, the average age of asthma patients was 44.2 years, 67.5 years in the COPD group, and 42.9 years in the healthy participant group. The distribution of participants by age categories is presented in Table 1. The chi-square test revealed a statistically significant difference in the distribution of age categories, with a younger population (especially ages 18–28 years) comprising a larger portion of the asthma group participants (23.4%) and participants from the general population without obstructive respiratory disease (26.0%), while the majority of participants diagnosed with COPD belonged to the age groups of 59–68 years (36.4%) and 69–80 years (51.5%). The sample’s gender distribution was evenly distributed among participants, with 51.1% of participants being female and 48.9% of participants being male. There was no statistically significant difference in the gender distribution of the three sample groups (Table 1). A statistically significant difference was observed when the distribution of participants by formal education level was analyzed in connection to the existence of asthma, COPD, and among individuals who are healthy (Table 1). Completed secondary education was the most common level of formal education in all three compared groups; however, it is noticeable that elementary education was more common among participants with COPD (30.3%) compared to those with asthma (10.6%) and healthy participants (3.0%). Moreover, participants with completed elementary or integrated academic studies (22.0%), as well as those with master’s or doctoral studies, were more represented among participants who do not suffer from obstructive respiratory diseases. In the analysis of the distribution of participants by employment status and monthly income level, the chi-square test results showed that a statistically significant difference existed among the examined groups (Table 1). Healthy participants and those with asthma were mostly employed, with monthly incomes ranging from 60,001 to 100,000 RSD (approximately 550–900 USD), unlike participants with COPD, who were predominantly retirees with monthly incomes between 30,000 and 60,000 dinars (approximately 270–550 USD). Analyzing the marital status of participants with asthma, COPD, and the healthy part of the sample, a statistically significant difference was found (Table 1): almost half of the asthma patients (40.4%) were single, while the majority of those with COPD (63.6%) and healthy participants (56.0%) were married or living with a partner. The largest number of participants from all three groups resided in urban areas, and the difference among the groups in this regard was not statistically significant (Table 1). 

Comorbidities were present in 28.9% of the total study sample. A notable 69.7% of COPD patients had associated diseases, while the frequency of comorbid conditions was significantly lower in the asthma group (27.7%) and in participants without obstructive respiratory diseases (16.0%), with these differences being statistically significant (χ^2^ = 34.87, df = 2, *p* < 0.001). Arterial hypertension was the most prevalent comorbidity in the whole sample, with cardiac arrhythmias, diabetes mellitus, and Hashimoto’s thyroiditis following closely behind. In order to determine if the related diseases’ frequencies in the three groups under comparison were similar, the chi-square test was employed. As demonstrated in Table 2, the findings indicated that arterial hypertension was a comorbidity that was statistically substantially more common in COPD patients (51.5%) compared to the asthma patients (17.0%) and healthy individuals in the sample (5.0%). Additionally, there was a statistically significant higher prevalence of diabetes mellitus in COPD (24.2%) compared to asthma patients (10.6%).

The analysis of responses from Group A participants revealed data suggesting that they were mostly long-term patients, with almost half of them having experienced previous hospitalizations due to exacerbations of their underlying conditions (although there were no intergroup differences, as shown in Table 3). Regarding the frequency of hospitalizations, there were no statistically significant differences between patients with COPD and those with asthma. A remarkable 90.0% of the surveyed patients reported regularly taking their medication. As before, no statistically significant differences were found among the observed groups of participants (Table 3).

### 3.2. Assessment of Asthma and COPD Control

Patients with asthma scored an average of 17.31 (±5.67) on the ACT, whereas those with COPD achieved an average of 19.09 (±9.45) on the CAT. The average scores that participants received on these surveys were thoroughly analyzed and are interpreted in Table 4.

### 3.3. Adherence with Inhalation Therapy

Participants evaluated their adherence with inhalation therapy using a four-point Likert scale (mostly/often—3 points, occasionally—2 points, seldom—1 point, and never—0 points for all items, except for items No. 4 and 8, which are reverse-scored), with a higher score indicating lower adherence. Patients with COPD and asthma appeared to have somewhat low adherence with inhaler medication, according to the composite score (M = 2.27; Table 5). According to an analysis of the questionnaire’s results, the item, “Do you report all adverse effects that you notice during inhalation therapy to your prescribing physician” (M = 0.56), had the lowest score, indicating the highest level of adherence in this area. The item, “Does information about possible adverse effects of inhalation therapy that you come across in the media (journals, TV, Internet, etc.) influence you to change the prescribed regimen without prior consultation with the prescribing physician” (M = 2.96), received the highest score, indicating the lowest level of adherence in this area. After the summary of the results from the respondents with COPD and asthma (Table 5), an independent sample *t*-test was performed to examine for any statistically significant variations in adherence between these two participant groups. The test results indicated a statistical significance level higher than *p* = 0.05. Therefore, it can be concluded that there were no statistically significant differences between the two groups analyzed. More specifically, responders with COPD and asthma demonstrated similar levels of adherence with inhalation therapy. Furthermore, as shown in Table 6, adherence to inhalation therapy among participants with different levels of asthma control and impact of COPD on patients’ life was similar, i.e., there was no statistically significant difference.

The Cronbach’s alpha coefficient, a frequently used test method for assessing internal consistency and reliability, was utilized in this study to evaluate the reliability of the adherence questionnaire. The individual items in this questionnaire had reliability values ranging from α = 0.306 to α = 0.612. Considering acceptable Cronbach’s alpha values remain above 0.70, with α = 0.468, the overall reliability of the AITQ was under the acceptable level. Despite this, the obtained reliability indicator value clearly demonstrated that this newly developed questionnaire is well-constructed metrically, but it requires supplementation with additional items.

### 3.4. Attitudes toward Inhalation Therapy

Participants’ attitudes toward inhalation therapy were assessed based on their selection of one of the offered responses on a five-point Likert scale (strongly disagree—1 point, disagree—2 points, neither agree nor disagree—3 points, agree—4 points, or strongly agree—5 points) to statements listed in the questionnaire. The questionnaire items were created based on the most frequently stated unfavorable attitudes and misconceptions about inhalation therapy in the literature, with only items 9, 12, 14, 17, and 18 being positively formed. A higher score on the item implies stronger agreement with the statement. Participants showed the highest level of agreement with statement No. 12: “If necessary, I would use the inhaler in front of others without feeling discomfort or shame about using this type of medication” (M = 4.40), followed by statement No. 18: “The propellant gas in pressurized aerosols used today is completely safe for health and does not harm the atmosphere” (M = 3.45), and statement No. 14: “Medications from most of the inhalers available on the market today do not affect the course of infection, so they can be used freely even during respiratory infections” (M = 3.29). The statement No. 10: “When using inhalers, medication should never be deeply inhaled, as the delivery of medication deep into the respiratory tract and lungs can cause a severe asthma attack”, received the lowest level of agreement from participants (M = 1.83). All of the questionnaire’s items had satisfactory reliability, with values ranging from α = 0.794 to α = 0.750 (Table 7). The overall reliability of the scale was α = 0.767.

A one-way analysis of variance (ANOVA) was conducted to examine whether the three sample groups (patients diagnosed with asthma, patients diagnosed with COPD, and healthy members of the general population) significantly differed in their attitudes toward the use of inhaler therapy (Table 7). Post-hoc comparisons were tested using the Tukey test. Statistically significant differences were obtained for the following items:

q1—Healthy participants more strongly agree with this statement compared to participants with asthma.

q2—The Tukey test did not confirm this difference.

q5—Healthy participants more strongly agree with this statement compared to participants with asthma.

q6—Healthy participants more strongly agree with this statement compared to both participants with asthma and COPD.

q8—Participants with COPD and healthy participants more strongly agree with this statement compared to patients with asthma.

q10—Healthy participants more strongly agree with this statement compared to participants with asthma.

q11—Healthy participants more strongly agree with this statement compared to participants with asthma.

q13—Participants with COPD more strongly agree with this statement compared to participants with asthma. 

q18—Healthy participants more strongly agree with this statement compared to participants with COPD.

q20—Participants with COPD and healthy participants more strongly agree with this statement compared to participants with asthma. 

## 4. Discussion

Inadequate treatment of obstructive pulmonary diseases may lead to a variety of consequences, including death due to respiratory failure [26]. While appropriate application of inhalation therapy is required for satisfactory control of asthma and COPD, research indicates that patients frequently do not fully adhere to the directions for using this type of medication. Immediate causes of poor adherence include denial of chronic illness, insufficient patient education, and numerous misconceptions regarding the supposed adverse effects of inhalation drugs [26]. This problem has been globally present in the treatment of pulmonary patients for decades, and nowadays it is becoming increasingly significant as a result of the rapid propagation of false, unconfirmed, or partially true information via the Internet and social media. The recent Infodemia phenomenon during the COVID-19 pandemic underlined the need for more attention from the professional community and health officials to this issue [27].

Similar to previous studies [28,29], the results of our study confirmed that asthma is predominantly a disease of younger individuals, while COPD is more prevalent among the older population. Analyzing the data on the participants’ formal education, it was obvious that individuals with asthma were generally more educated than those with COPD. These data are consistent with the generally accepted view that the influence of factors contributing to the onset of asthma cannot be completely avoided. On the other hand, the progression of COPD is typically strongly connected to long-term cigarette smoking, reflecting deliberate and voluntary exposure to this detrimental factor. This also implies that health literacy is typically low among these individuals [1,5,30].

Both asthma and COPD are diseases associated with a large number of potential comorbidities [31,32]. In our sample, comorbid disorders were significantly more prevalent in the COPD group, affecting up to 69.7% of participants, whereas related diseases were documented in more than twice as few asthma patients (27.7%). Analyzing the comorbidity profile of the study participants, it can be concluded that both arterial hypertension and diabetes mellitus, as the most common comorbidities in the overall A group of participants, statistically significantly appeared more frequently in those with COPD than in those with asthma. Overall, smoking is a considerable risk factor for the development of numerous comorbidities, particularly cardiovascular ones [33]. Considering that this harmful habit is more common in individuals with COPD, such a result was expected.

One of the important characteristics of obstructive respiratory diseases is their chronic course with a high frequency of exacerbations [1,5,34,35]. Accordingly, a significant part of the A group in our sample consisted of long-term patients in whom exacerbations of the underlying disease occurred in more than 50% of cases. However, in 42.6% of asthma patients in our sample, asthma was classified as well controlled based on the achieved ACT score. For comparison, a recent Portuguese study [36] found that over half of the participants’ asthma was rated as only partially controlled, with none having well-controlled asthma at the time of the study, indicating a good approach to asthma treatment in the individuals included in our research. At the same time, the analysis of results obtained from part of the A group participants suffering from COPD indicated a moderate to high impact of this disease on their health and daily lives: the average CAT questionnaire score in our study was 19.09, slightly higher than in the POPE study [37] conducted on COPD patients in Central and Eastern Europe, where the average score was 17.5. Based on this data, it can be concluded that COPD in Serbia similarly affects the health status and daily life of patients.

No medication can demonstrate full therapeutic efficacy if it is not taken regularly, at the therapeutic dose, and as prescribed [38]. From the perspective of medication adherence, inadequate patient cooperation in the therapeutic process likely represents the most significant obstacle in the clinical treatment of asthma and COPD [15]. Previous studies implied that taking medication is not a high priority for a segment of the affected population due to denial of the presence of a chronic illness. Often, such patients do not understand or accept the need for long-term therapy aimed at improving lung function and preventing exacerbations, as it has long been observed that those with chronic diseases are less aware of their limitations [20]. Moreover, a large number of patients believe that diseases such as asthma are largely psychologically caused. Consequently, they often believe that reducing symptom intensity will occur simply through positive thinking, or if they think positively about feeling better [39]. The results of our study align with the previously mentioned findings: although the majority of asthma and COPD patients in our sample believed they regularly adhere to the prescribed inhalation therapy (as much as 90% of the A group respondents), the lower score on the newly created AITQ clearly indicated low adherence in both groups of respondents, with no statistically significant differences in adherence between asthma and COPD patients. Although the questionnaire’s reliability indicator (α = 0.468) was below the acceptable level through statistical testing, the adherence results clearly demonstrated the issue’s current relevance among patients with obstructive respiratory diseases.

On the other hand, the observation that there were no significant differences in treatment adherence between asthma and COPD patients despite notable disparities in socioeconomic and demographic factors, such as education, salary, and marital status, warrants careful consideration. While these variables are known to influence healthcare behaviors and treatment adherence in chronic disease management, the absence of a discernible impact on adherence in our study population may be attributed to several factors. It is possible that factors beyond socioeconomic and demographic characteristics play a more prominent role in influencing treatment adherence in patients with obstructive pulmonary diseases, such as symptom severity, perceived treatment efficacy, and the presence of comorbidities. Additionally, psychosocial factors, such as social support, healthcare provider–patient communication, and access to healthcare resources, may play a mediating role in mitigating the impact of socioeconomic disparities on treatment adherence. However, we found the lack of statistically significant differences regardless of the asthma control measured by the ACT or the impact of COPD on patients’ life measured by the CAT, which may seem unexpected. It is possible that other confounding variables may have influenced these results. For instance, the sub-group samples may have been relatively small, limiting the statistical power to detect differences. Additionally, it is possible that patients with more severe disease tend to exhibit higher adherence to their treatment regimen. However, due to the increased severity of their condition, they may consequently experience poorer ACT or CAT scores. 

Unlike AITQ, the statistical testing revealed satisfactory reliability of the newly constructed ITAQ (α = 0.767). After examining respondents’ attitudes from both sample groups regarding inhalation therapy, it was found that respondents without obstructive respiratory disease (Group B) were more likely than respondents with asthma to have misconceptions about the treatment. This is somewhat expected, as individuals who use these medications are generally more informed compared to those who do not have such experience. Analysis of the responses from both groups of respondents in the sample revealed that misconceptions most commonly relate to the efficacy, side effects, and addictive potential of medications administered through inhalation, highlighting the need for the education of general population on the benefits and risks of inhalation therapy. The results obtained were very similar to those found in previous studies worldwide, including more recent ones [20,36,40]. This suggests that the issue of inadequate information, accessibility to incorrect information, and inadequate education of asthma and COPD patients is a pressing problem that requires urgent attention. The results of the research showed that misconceptions about inhalation therapy are more prevalent among respondents with COPD compared to those with asthma, which can be attributed to the generally lower level of health literacy in this segment of the patient population. This finding also underscores the unique challenges faced by individuals with COPD, who may grapple with complex treatment regimens and progressive disease severity. The identification of misconceptions and variations in attitudes toward inhalation therapy among different patient populations has important implications for healthcare providers and policymakers. Addressing and correcting misconceptions about inhalation therapy through targeted education and counseling interventions is paramount to promoting treatment adherence and optimizing health outcomes, particularly among individuals with COPD who may be more prone to harboring misconceptions. Finally, in an era characterized by the availability of various information related to the treatment of these diseases, safety information grounded in pharmacovigilance activities should be easily accessible and serve as the most dependable source regarding the safety of inhalation drugs [16].

The utilization of two novel questionnaires that were used in our study in clinical practice harbors the potential to ameliorate patient outcomes through the optimization of adherence to inhalation therapy and the proactive addressing of barriers to treatment adherence. These tools facilitate the identification of patients at heightened risk of poor adherence to inhalation therapy. This identification enables healthcare providers to implement additional interventions for those patients requiring supplementary support in adhering to their treatment regimen. In light of the insights garnered from these questionnaires, healthcare providers possess the capability to tailor treatment plans to align more closely with individual patient needs. Furthermore, there exists an opportunity to place greater emphasis on patient education regarding the significance of inhalation therapy and to address any misconceptions or concerns they may harbor. Indeed, educated patients are more inclined to actively engage in their treatment and adhere to prescribed regimens. The routine utilization of these tools enables healthcare providers to monitor adherence to inhalation therapy longitudinally and to guide subsequent treatment decisions accordingly. For instance, in scenarios where patients have not achieved the desired level of disease control despite demonstrating high adherence to prescribed therapy, healthcare providers can contemplate adjustments such as changing the prescribed inhaler or molecule.

Several study limitations should be addressed as well. Firstly, it is important to note that due to the constraints of conducting research in a single healthcare center and the inherent challenges in recruiting participants with specific respiratory conditions, such as COPD and asthma, the sample size was ultimately limited. As such, the generalizability of the findings to the wider population should be interpreted with caution. The inclusion of a larger and more diverse sample, particularly encompassing patients from various healthcare centers, could potentially furnish more comprehensive and accurate insights into adherence behaviors, disease control, and misconceptions pertaining to inhalation therapy among individuals with obstructive pulmonary diseases. Secondly, the possibility of response bias must be acknowledged, as patients may not have been entirely forthcoming or truthful in their responses while completing the surveys. Moreover, the reliability indicator of the AITQ fell below the acceptable threshold, indicating potential shortcomings in its psychometric properties. As such, future research endeavors should prioritize the refinement and enhancement of the instrument, possibly through the incorporation of additional questions pertaining to adherence to inhalation therapy. Future studies could also explore in more depth the impact of smoking status and other sociodemographic factors of interest, such as age, sex, and educational level, on adherence to inhalation therapy. 

## 5. Conclusions

Although the majority of patients with asthma and COPD in our survey felt they regularly adhered to their prescribed inhalation therapy, it was evident that adherence in both categories of respondents was low. Importantly, there were no statistically significant variations in adherence between patients with asthma and COPD. Most patients with asthma demonstrated satisfactory disease control, whereas the majority of patients with COPD exhibited a moderate to high impact of COPD on their health status and daily life. Our study found that misconceptions about the use of inhalation therapy were more common among healthy participants without obstructive respiratory disorders than among those with asthma. The most common misunderstandings in the assessed population were about inhalation medicines’ effectiveness, adverse effects, and addictive potential. In addition, participants with COPD had greater misconceptions regarding inhalation therapy than those with asthma, emphasizing the importance of organized and continuous health education programs in this patient population. Finally, the outcomes of this study highlighted the necessity of increasing health literacy and awareness, not just among affected individuals but also in the general population, confirming that this is also an important public health challenge. 

## Figures and Tables

**Table 1:** Sociodemographic analysis of the study sample.

	All		Group						χ^2^	df	*p*
			Asthma		COPD		Healthy				
	f	%	f	%	f	%	f	%			
Age											
18–28 years	37	20.6%	11	23.4%	0	0.0%	26	26.0%	84.94	10	0.000
29–38 years	16	8.9%	6	12.8%	1	3.0%	9	9.0%			
39–48 years	37	20.6%	12	25.5%	0	0.0%	25	25.0%			
49–58 years	40	22.2%	7	14.9%	3	9.1%	30	30.0%			
59–68 years	26	14.4%	7	14.9%	12	36.4%	7	7.0%			
69–80 years	24	13.3%	4	8.5%	17	51.5%	3	3.0%			
Gender											
Male	88	48.9%	18	38.3%	21	63.6%	49	49.0%	4.98	2	0.083
Female	92	51.1%	29	61.7%	12	36.4%	51	51.0%			
Formal Education Level											
Elementary School	18	10.0%	5	10.6%	10	30.3%	3	3.0%	30.26	8	0.000
High School	96	53.3%	29	61.7%	18	54.5%	49	49.0%			
Elementary or Integrated Academic Studies	28	15.6%	5	10.6%	1	3.0%	22	22.0%			
Vocational Studies	11	6.1%	3	6.4%	2	6.1%	6	6.0%			
Master’s/Doctorate	27	15.0%	5	10.6%	2	6.1%	20	20.0%			
Employment Status											
Employed	119	66.1%	30	63.8%	3	9.1%	86	86.0%	93.37	4	0.000
Unemployed	19	10.6%	7	14.9%	2	6.1%	10	10.0%			
Retired	42	23.3%	10	21.3%	28	84.8%	4	4.0%			
Monthly Income											
Less than 30,000 RSD	36	20.0%	11	23.4%	12	36.4%	13	13.0%	14.87	6	0.021
30,000–60,000 RSD	57	31.7%	14	29.8%	13	39.4%	30	30.0%			
60,001–100,000 RSD	69	38.3%	19	40.4%	7	21.2%	43	43.0%			
More than 100,000 RSD	18	10.0%	3	6.4%	1	3.0%	14	14.0%			
Marital Status											
Single	49	27.2%	19	40.4%	1	3.0%	29	29.0%	22.13	8	0.005
Living with partner	15	8.3%	3	6.4%	3	9.1%	9	9.0%			
Married	98	54.4%	21	44.7%	21	63.6%	56	56.0%			
Divorced	8	4.4%	3	6.4%	3	9.1%	2	2.0%			
Widowed	10	5.6%	1	2.1%	5	15.2%	4	4.0%			
Residential Area											
Urban	112	62.2%	34	72.3%	19	57.6%	59	59.0%	9.28	4	0.055
Rural	33	18.3%	8	17.0%	10	30.3%	15	15.0%			
Suburban	35	19.4%	5	10.6%	4	12.1%	26	26.0%			

f = Frequency; % = percentage; χ^2^ = chi-square test; df = degrees of freedom; *p* = statistical significance.

**Table 2:** Analysis of comorbidities in the examined sample.

		All		Group						χ^2^	df	*p*
				Asthma		COPD		Healthy				
		f	%	f	%	f	%	f	%			
Arterial Hypertension	Yes	30	16.7%	8	17.0%	17	51.5%	5	5.0%	38.66	2	0.000
	No	150	83.3%	39	83.0%	16	48.5%	95	95.0%			
	Total	180	100.0%	47	100.0%	33	100.0%	100	100.0%			
Diabetes Mellitus	Yes	15	8.3%	5	10.6%	8	24.2%	2	2.0%	16.51	2	0.000
	No	165	91.7%	42	89.4%	25	75.8%	98	98.0%			
	Total	180	100.0%	47	100.0%	33	100.0%	100	100.0%			
Tachycardia	Yes	2	1.1%	0	0.0%	0	0.0%	2	2.0%	1.62	2	0.445
	No	178	98.9%	47	100.0%	33	100.0%	98	98.0%			
	Total	180	100.0%	47	100.0%	33	100.0%	100	100.0%			
Thyroiditis (unspecified)	Yes	1	0.6%	0	0.0%	0	0.0%	1	1.0%	0.80	2	0.669
	No	179	99.4%	47	100.0%	33	100.0%	99	99.0%			
	Total	180	100.0%	47	100.0%	33	100.0%	100	100.0%			
Hashimoto’s Thyroiditis	Yes	7	3.9%	2	4.3%	2	6.1%	3	3.0%	0.64	2	0.724
	No	173	96.1%	45	95.7%	31	93.9%	97	97.0%			
	Total	180	100.0%	47	100.0%	33	100.0%	100	100.0%			
Arthritis	Yes	2	1.1%	1	2.1%	0	0.0%	1	1.0%	0.82	2	0.662
	No	178	98.9%	46	97.9%	33	100.0%	99	99.0%			
	Total	180	100.0%	47	100.0%	33	100.0%	100	100.0%			
Arrhythmias	Yes	3	1.7%	2	4.3%	0	0.0%	1	1.0%	2.75	2	0.253
	No	177	98.3%	45	95.7%	33	100.0%	99	99.0%			
	Total	180	100.0%	47	100.0%	33	100.0%	100	100.0%			
Ulcerative Colitis	Yes	2	1.1%	0	0.0%	1	3.0%	1	1.0%	1.65	2	0.439
	No	178	98.9%	47	100.0%	32	97.0%	99	99.0%			
	Total	180	100.0%	47	100.0%	33	100.0%	100	100.0%			
Bronchiectasis	Yes	2	1.1%	0	0.0%	1	3.0%	1	1.0%	1.65	2	0.439
	No	178	98.9%	47	100.0%	32	97.0%	99	99.0%			
	Total	180	100.0%	47	100.0%	33	100.0%	100	100.0%			
Malignancy	Yes	2	1.1%	0	0.0%	1	3.0%	1	1.0%	1.65	2	0.439
	No	178	98.9%	47	100.0%	32	97.0%	99	99.0%			
	Total	180	100.0%	47	100.0%	33	100.0%	100	100.0%			
Osteoporosis	Yes	2	1.1%	0	0.0%	1	3.0%	1	1.0%	1.65	2	0.439
	No	178	98.9%	47	100.0%	32	97.0%	99	99.0%			
	Total	180	100.0%	47	100.0%	33	100.0%	100	100.0%			
Hyperlipidemia	Yes	2	1.1%	0	0.0%	1	3.0%	1	1.0%	1.65	2	0.439
	No	178	98.9%	47	100.0%	32	97.0%	99	99.0%			
	Total	180	100.0%	47	100.0%	33	100.0%	100	100.0%			

f = Frequency; % = percentage; χ^2^ = chi-square test; df = degrees of freedom; *p* = statistical significance.

**Table 3:** Analysis of data related to the course of the disease in participants from Group A (asthma and COPD patients).

	All		Group				χ^2^	df	*p*
			Asthma		COPD				
	f	%	f	%	f	%			
Obstructive respiratory disease diagnosed before									
0.1–3 years	23	31.1%	17	38.6%	6	20.0%	14.18	3	0.003
3.1–5 years	14	18.9%	5	11.4%	9	30.0%			
5.1–10 years	14	18.9%	4	9.1%	10	33.3%			
More than 10 years	23	31.1%	18	40.9%	5	16.7%			
Previous hospitalizations due to asthma or COPD									
Yes	35	43.8%	20	42.6%	15	45.5%	0.07	1	0.797
No	45	56.3%	27	57.4%	18	54.5%			
Number of previous hospitalizations due to asthma or COPD									
0	45	56.3%	27	57.4%	18	54.5%	0.36	2	0.835
1	10	12.5%	5	10.6%	5	15.2%			
2+	25	31.3%	15	31.9%	10	30.3%			
Regular use of the recommended inhalation therapy									
Yes	72	90.0%	43	91.5%	29	87.9%	0.28	1	0.596
No	8	10.0%	4	8.5%	4	12.1%			

f = Frequency; % = percentage; χ^2^ = chi-square test; df = degrees of freedom; *p* = statistical significance.

**Table 4:** Interpretation of the obtained ACT and CAT results.

		f	%
ACT categories	Well-controlled asthma	17	36.2%
	Not well-controlled asthma	10	21.3%
	Very poorly controlled asthma	20	42.6%
CAT categories	Low impact of COPD on patient’s life	7	21.2%
	Mild impact of COPD on patient’s life	11	33.3%
	High impact of COPD on patient’s life	10	30.3%
	Very high impact of COPD on patient’s life	5	15.2%

f = Frequency; % = percentage.

**Table 5:** Descriptive indicators and reliability of the AITQ.

	Item	Group	M (±SD)	t	df	*p*	α
q1	Do you believe that adhering to inhalation therapy as prescribed by the physician is not always in line with your health needs?	Total	2.43 (0.96)	−0.698	78	0.487	0.397
		Asthma	2.36 (0.92)				
		COPD	2.52 (1.03)				
q2	In the last three months, have there been situations where you have independently altered the prescribed therapeutic regimen (dosage, frequency, etc.) based on your own assessments without consulting a physician or other competent healthcare professional?	Total	2.44 (0.90)	0.110	78	0.913	0.348
		Asthma	2.45 (0.90)				
		COPD	2.42 (0.90)				
q3	In the last three months, have there been periods when you did not take the prescribed inhalation therapy at all because you felt well, without reporting this to the prescribing physician?	Total	2.40 (1.00)	−0.861	78	0.392	0.306
		Asthma	2.32 (1.02)				
		COPD	2.52 (0.97)				
q4	Do you report all adverse effects that you notice during inhalation therapy to your prescribing physician?	Total	0.56 (1.09)	−0.091	78	0.928	0.612
		Asthma	0.55 (1.06)				
		COPD	0.58 (1.15)				
q5	In the last three months, have you interrupted your inhalation therapy because you felt unwell after using it?	Total	2.84 (0.51)	1.168	78	0.247	0.460
		Asthma	2.89 (0.43)				
		COPD	2.76 (0.61)				
q6	Does complying with the physician’s prescription for inhalation therapy interfere with your comfort and daily life activities?	Total	2.73 (0.75)	0.889	78	0.376	0.445
		Asthma	2.79 (0.69)				
		COPD	2.64 (0.82)				
q7	Do you unintentionally forget to take the prescribed inhalation therapy?	Total	2.29 (0.87)	0.384	78	0.702	0.381
		Asthma	2.32 (0.91)				
		COPD	2.24 (0.83)				
q8	Do you take the prescribed inhalation therapy even when you are away from home due to certain circumstances?	Total	1.19 (1.20)	−1.487	78	0.141	0.526
		Asthma	1.02 (1.17)				
		COPD	1.42 (1.23)				
q9	Does information about possible adverse effects of inhalation therapy that you come across in the media (journals, TV, Internet, etc.) influence you to change the prescribed regimen without prior consultation with the prescribing physician?	Total	2.96 (0.25)	−0.216	78	0.830	0.444
		Asthma	2.96 (0.29)				
		COPD	2.97 (0.17)				
q10	In the last three months, have you changed the prescribed regimen of inhalation therapy based on information received from others (not from the prescribing physician) without first verifying its accuracy with the physician?	Total	2.90 (0.44)	−0.671	78	0.504	0.408
		Asthma	2.87 (0.54)				
		COPD	2.94 (0.24)				
	Composite score	Total	2.27 (0.35)	−0.582	78	0.563	0.468
		Asthma	2.25 (0.35)				
		COPD	2.30 (0.36)				

M = mean; SD = standard deviation; t = *t*-test; df = degrees of freedom; *p* = statistical significance; α = Cronbach’s alpha coefficient.

**Table 6:** The correlation between ACT and CAT categories and adherence to inhalation therapy.

		Adherence		F	*p*
		M	SD		
ACT categories	Well-controlled asthma	2.35	0.24	1.544	0.337
	Not well-controlled asthma	2.01	0.31		
	Very poorly controlled asthma	2.29	0.40		
CAT categories	Low impact of COPD on patient’s life	2.46	0.24	1.968	0.141
	Mild impact of COPD on patient’s life	2.37	0.37		
	High impact of COPD on patient’s life	2.26	0.30		
	Very high impact of COPD on patient’s life	2.00	0.48		

M = mean; SD = standard deviation, F = ANOVA, *p* = statistical significance.

**Table 7:** Descriptive indicators and reliability of the ITAQ.

	Item	Group	M (±SD)	F	*p*	α
q1	Inhalation therapy is not necessarily an essential method for treating and controlling asthma or chronic obstructive pulmonary disease (COPD) in all patients—good results can often be achieved solely through the use of oral medications	Total	2.57 (1.16)	8.017	0.000	0.771
		Asthma	2.02 (1.03)			
		COPD	2.61 (1.17)			
		Healthy	2.81 (1.13)			
q2	Inhalation therapy is not safer compared to orally administered medications, as drugs taken in this manner also have a strong effect on the entire body	Total	2.57 (1.17)	3.142	0.046	0.761
		Asthma	2.21 (1.14)			
		COPD	2.79 (1.14)			
		Healthy	2.66 (1.17)			
q3	Inhalation therapy administered via a nebulizer (home or hospital type) is safer than therapy using pressurized metered-dosed, soft mist, or dry powder inhalers, as it much less frequently leads to the occurrence of adverse reactions	Total	2.97 (1.06)	2.415	0.092	0.776
		Asthma	2.79 (1.06)			
		COPD	2.76 (1.12)			
		Healthy	3.12 (1.02)			
q4	Prescribing inhalers often indicates the severity of obstructive respiratory disease, as they are used only in patients with the most severe forms of asthma or COPD	Total	2.56 (1.37)	2.661	0.073	0.763
		Asthma	2.30 (1.27)			
		COPD	2.30 (1.49)			
		Healthy	2.77 (1.35)			
q5	Initiating inhaler therapy should be delayed as long as possible, as once started, it must be used for a lifetime	Total	2.25 (1.37)	4.250	0.016	0.750
		Asthma	1.83 (1.20)			
		COPD	2.09 (1.26)			
		Healthy	2.50 (1.43)			
q6	As soon as a patient feels better, they should stop using inhalers to prevent becoming addicted to them	Total	2.28 (1.33)	6.140	0.003	0.758
		Asthma	1.87 (1.19)			
		COPD	1.94 (1.37)			
		Healthy	2.58 (1.32)			
q7	Nebulizer therapy is the preferred method of treating childhood asthma because inhaler use during childhood might cause difficulties in growth and development	Total	2.48 (1.17)	0.331	0.719	0.766
		Asthma	2.60 (1.26)			
		COPD	2.39 (1.14)			
		Healthy	2.46 (1.14)			
q8	If necessary, I would always prefer to take medications orally (in the form of tablets or capsules), as all inhalers result in some degree of lasting damage to heart function	Total	2.57 (1.21)	5.968	0.003	0.750
		Asthma	2.11 (0.98)			
		COPD	3.00 (1.35)			
		Healthy	2.64 (1.21)			
q9	The use of inhalers is safe during pregnancy, these medications cannot lead to fetal damage	Total	3.18 (1.06)	2.758	0.066	0.790
		Asthma	3.15 (1.02)			
		COPD	2.82 (1.01)			
		Healthy	3.31 (1.07)			
q10	The medication from the inhaler should never be deeply inhaled, as its delivery deep into the respiratory tract and lungs can cause a severe asthma/COPD attack	Total	1.83 (1.09)	7.777	0.001	0.754
		Asthma	1.40 (0.90)			
		COPD	1.64 (1.22)			
		Healthy	2.10 (1.05)			
q11	Due to the patient’s body gradually becoming accustomed to the inhalers, it is important to use caution when determining how often and how long to use them in order to achieve the intended therapeutic effect	Total	2.88 (1.26)	9.480	0.000	0.754
		Asthma	2.26 (1.19)			
		COPD	2.88 (1.27)			
		Healthy	3.18 (1.18)			
q12	If necessary, I would use the inhaler in front of others without feeling discomfort or shame about using this type of medication	Total	4.40 (1.12)	0.330	0.719	0.789
		Asthma	4.34 (1.24)			
		COPD	4.30 (1.29)			
		Healthy	4.46 (1.01)			
q13	In the case of an asthma or COPD attack, it is always better to administer the appropriate medication orally or by injection, as the effect of inhalers takes significantly longer to occur compared to these methods of administration	Total	2.73 (1.34)	4.018	0.020	0.763
		Asthma	2.36 (1.34)			
		COPD	3.21 (1.41)			
		Healthy	2.74 (1.28)			
q14	The majority of substances in registered inhalers do not change the course of infection, therefore they can be safely used even during respiratory infections	Total	3.29 (1.10)	0.110	0.896	0.794
		Asthma	3.34 (1.22)			
		COPD	3.33 (1.16)			
		Healthy	3.26 (1.02)			
q15	Medication administered through inhalation has a weaker effect, hence the dosage in inhalation preparations is always significantly higher than in oral preparations	Total	2.77 (1.09)	1.228	0.295	0.766
		Asthma	2.74 (1.13)			
		COPD	3.03 (1.13)			
		Healthy	2.69 (1.05)			
q16	In addition to having a weaker effect, inhalation therapy is also less comfortable for the patient compared to oral therapy, as it needs to be administered more frequently throughout the day	Total	2.53 (1.21)	1.629	0.199	0.753
		Asthma	2.28 (1.12)			
		COPD	2.52 (1.35)			
		Healthy	2.66 (1.19)			
q17	Inhalation therapy is equally effective in smokers, as smoking does not affect the quality of the therapeutic response to medications administered via inhalation	Total	2.14 (1.16)	0.504	0.605	0.773
		Asthma	2.00 (1.22)			
		COPD	2.24 (1.35)			
		Healthy	2.17 (1.06)			
q18	The propellant gas in pressurized inhalers used today is completely safe for health and does not harm the atmosphere	Total	3.45 (1.13)	4.084	0.018	0.789
		Asthma	3.23 (1.05)			
		COPD	3.12 (1.43)			
		Healthy	3.66 (1.03)			
q19	Allergic reactions following inhalation therapy are often much more severe compared to oral medication, as the drug directly penetrates into the lungs	Total	2.82 (1.08)	0.301	0.740	0.767
		Asthma	2.72 (1.17)			
		COPD	2.91 (1.04)			
		Healthy	2.83 (1.05)			
q20	There are not justified reasons to prescribe inhalation therapy so frequently to patients with asthma and COPD. This is likely due to the pressure imposed on physicians by pharmaceutical companies that produce these often-expensive medications	Total	2.77 (1.34)	7.793	0.001	0.756
		Asthma	2.19 (1.17)			
		COPD	3.30 (1.36)			
		Healthy	2.86 (1.32)			

M = mean; SD = standard deviation; F = ANOVA; *p* = statistical significance; α = Cronbach’s alpha coefficient.

## Data Availability

The data that support the findings of this study are available from the corresponding author upon reasonable request. The data are not publicly available due to privacy.

## References

[1] Global Initiative for Chronic Obstructive Lung Disease (GOLD) (2024). Global Strategy for the Diagnosis, Management, and Prevention of Chronic Obstructive Pulmonary Disease (2024 Report). https://goldcopd.org/2024-gold-report/.

[2] Christenson S.A., Smith B.M., Bafadhel M., Putcha N. (2022). Chronic obstructive pulmonary disease. Lancet.

[3] Vukoja M., Kopitovic I., Lazic Z., Milenkovic B., Stankovic I., Zvezdin B., Ilic A.D., Cekerevac I., Vukcevic M., Zugic V. (2019). Diagnosis and management of chronic obstructive pulmonary disease in Serbia: An expert group position statement. Int. J. Chronic Obstr. Pulm. Dis..

[4] Labaki W.W., Rosenberg S.R. (2020). Chronic Obstructive Pulmonary Disease. Ann. Intern. Med..

[5] Global Initiative for Asthma (2023). Global Strategy for Asthma Management and Prevention (2023 Update). https://www.ginasthma.org.

[6] Mims J.W. (2015). Asthma: Definitions and pathophysiology. Int. Forum Allergy Rhinol..

[7] Melani A.S. (2007). Inhalatory therapy training: A priority challenge for the physician. Acta Biomed..

[8] Billington C.K., Penn R.B., Hall I.P. (2017). β2 Agonists. Handb. Exp. Pharmacol..

[9] Vukoja M., Kopitovic I., Lazic Z., Milenkovic B., Stankovic I., Tomic-Spiric V., Zvezdin B., Hromis S., Cekerevac I., Ilic A. (2022). Diagnosis and treatment of adult asthma patients in Serbia: A 2022 experts group position statement. Expert. Rev. Respir. Med..

[10] Cardet J.C., Papi A., Reddel H.K. (2023). “As-Needed” Inhaled Corticosteroids for Patients with Asthma. J. Allergy Clin. Immunol. Pract..

[11] Melani A.S. (2021). Inhaler technique in asthma and COPD: Challenges and unmet knowledge that can contribute to suboptimal use in real life. Expert. Rev. Clin. Pharmacol..

[12] Ari A. (2015). Patient Education and Adherence to Aerosol Therapy. Respir. Care.

[13] Lareau S.C., Yawn B.P. (2010). Improving adherence with inhaler therapy in COPD. Int. J. Chronic Obstr. Pulm. Dis..

[14] Ilic M., Bjelic D., Javorac J., Tot Veres K., Zivanovic D., Kovačevic D., Maric N., Lalic N., Colic N., Stjepanovic M. (2023). Knowledge is the most powerful tool in the fight against tuberculosis. J. Infect. Dev. Ctries..

[15] Ulrik C.S., Backer V., Søes-Petersen U., Lange P., Harving H., Plaschke P.P. (2006). The Patient’s Perspective: Adherence or Non-adherence to Asthma Controller Therapy?. J. Asthma.

[16] Chan A., De Simoni A., Wileman V., Holliday L., Newby C.J., Chisari C., Ali S., Zhu N., Padakanti P., Pinprachanan V. (2022). Digital interventions to improve adherence to maintenance medication in asthma. Cochrane Database Syst. Rev..

[17] Chan P.W., DeBruyne J.A. (2000). Parental concern towards the use of inhaled therapy in children with chronic asthma. Pediatr. Int..

[18] Sulaiman S.P., Panicker V. (2017). Attitudes of patients with asthma on inhaler use—A cross-sectional study from South Kerala. J. Evid. Based Med. Healthc..

[19] Rubin B.K. (2015). Asthma myths, controversies, and dogma. Paediatr. Respir. Rev..

[20] Dumra H., Khanna A., Madhukar S.K., Lopez M., Gogtay J. (2022). Perceptions and Attitudes of Patients and Their Family Caregivers on Nebulization Therapy for COPD. Int. J. Chronic Obstr. Pulm. Dis..

[21] Ip K.I., Hon K.L., Tsang K.Y.C., Leung T.N.H. (2018). Steroid phobia, Chinese medicine and asthma control. Clin. Respir. J..

[22] Boulet L.P. (1998). Perception of the role and potential side effects of inhaled corticosteroids among asthmatic patients. Chest.

[23] Vrijens B., De Geest S., Hughes D.A., Przemyslaw K., Demonceau J., Ruppar T., Dobbels F., Fargher E., Morrison V., Lewek P. (2012). A new taxonomy for describing and defining adherence to medications. Br. J. Clin. Pharmacol..

[24] Milenković B., Dimić Janjić S., Kotur Stevuljević J., Kopitović I., Janković J., Stjepanović M., Vukoja M., Ristic S., Davicevic-Elez Z. (2020). Validation of Serbian version of chronic obstructive pulmonary disease assessment test. Vojnosanit. Pregl..

[25] Milenković B., Ristić S., Mirović D., Dimić Janjić S., Janković J., Đurđević N., Davicevic-Elez Z., Lukic T. (2019). Validation of the Serbian version of the Asthma Control Test. Vojnosanit. Pregl..

[26] Gare M.B., Godana G.H., Zewdu B. (2020). Knowledge, Attitude, and Practice Assessment of Adult Asthmatic Patients towards Pharmacotherapy of Asthma at Jimma University Specialized Hospital. EC Pulmonol. Respir. Med..

[27] Živanović D., Jovin V.M., Javorac J., Ilić M., Zelić P. (2021). Commentary: Registered adverse events following COVID-19 immunization in Serbia. Eur. Rev. Med. Pharmacol. Sci..

[28] Patadia M.O., Murrill L.L., Corey J. (2014). Asthma: Symptoms and presentation. Otolaryngol. Clin. N. Am..

[29] Buist A.S., McBurnie M.A., Vollmer W.M., Gillespie S., Burney P., Mannino D.M., Menezes A.M., Sullivan S.D., A Lee T., Weiss K.B. (2007). International variation in the prevalence of COPD (the BOLD Study): A population-based prevalence study. Lancet.

[30] Omori H., Yoshimoto D., Kumar M., Goren A. (2016). Prevalence, awareness, characteristics, and health outcomes associated with COPD at-risk status among adults in Japan. Expert. Rev. Pharmacoecon. Outcomes Res..

[31] Cavaillès A., Brinchault-Rabin G., Dixmier A., Goupil F., Gut-Gobert C., Marchand-Adam S., Meurice J.-C., Morel H., Person-Tacnet C., Leroyer C. (2013). Comorbidities of COPD. Eur. Respir. Rev..

[32] Cazzola M., Rogliani P., Ora J., Calzetta L., Matera M.G. (2022). Asthma and comorbidities: Recent advances. Pol. Arch. Intern. Med..

[33] Ambrose J.A., Barua R.S. (2004). The pathophysiology of cigarette smoking and cardiovascular disease: An update. J. Am. Coll. Cardiol..

[34] Castillo J.R., Peters S.P., Busse W.W. (2017). Asthma Exacerbations: Pathogenesis, Prevention, and Treatment. J. Allergy Clin. Immunol. Pract..

[35] Javorac J., Živanović D., Ilić M., Kašiković Lečić S., Milenković A., Dragić N., Bijelović S., Savić N., Vereš K.T., Smuđa M. (2023). The influence of air pollution on non-infectious hospitalizations for severe acute exacerbations of chronic obstructive pulmonary disease: A time-series from Serbia. Atmosphere.

[36] Camarinha C., Fernandes M., Alarc Úo V., Franco J., Mana.°as M.E., B Írbara C., Nicola P.J. (2023). Determinants associated with uncontrolled asthma in Portugal: A national population-based study. Pulmonology.

[37] Miravitlles M., Koblizek V., Esquinas C., Milenkovic B., Barczyk A., Tkacova R., Somfay A., Zykov K., Tudoric N., Kostov K. (2019). Determinants of CAT (COPD Assessment Test) scores in a population of patients with COPD in central and Eastern Europe: The POPE study. Respir. Med..

[38] Živanović D., Mijatović Jovin V., Javorac J., Kvrgić S., Rašković A., Stojkov S., Lečić S.K., Savić N., Kralj M., Milenković A. (2022). Measuring pharmacovigilance knowledge and attitudes among healthcare sciences students: Development and validation of a universal questionnaire. Eur. Rev. Med. Pharmacol. Sci..

[39] DiMatteo M.R., Haskard K.B., Williams S.L. (2007). Health beliefs, disease severity, and patient adherence: A meta-analysis. Med. Care.

[40] Hui R.W.H. (2020). Inhaled corticosteroid-phobia and childhood asthma: Current understanding and management implications. Paediatr. Respir. Rev..

